# Notice of the Spotted Fever, as It Occurred at Newport, Rhode Island, in the Months of January, February, March, and April, 1863, with a History of the Disease, Its Symptoms, Diagnosis, and Treatment

**Published:** 1864-02

**Authors:** Philip S. Wales

**Affiliations:** Surgeon U.S.N.


### Selections.

### NOTICE OF THE SPOTTED FEVER,

### AS IT OCCURRED AT NEWPORT, RHODE ISLAND, IN THE MONTHS OF JAN., FEB., MARCH, AND APRIL, 1863, WITH A HISTORY OF THE DISEASE, ITS SYMPTOMS, DIAGNOSIS, AND TREATMENT.

By PHILIP S. WALES, M.D., Surgeon U.S.N.

During the past winter, several sections of our country have been visited by a disease of a febrile character, very little known and marked by peculiarities clearly distinguishing it from those fevers ordinarily met with. To some of the localities it appears to be an entire stranger, as in Philadelphia, for instance, where Dr. Gerhard assures us it never was witnessed before. The two epidemics of negro fever occurring in that city, the first in 1820-21, the second in 1848, were entirely different in their course and symptoms from the one under consideration, yet recognizing some affinity with it in being blood diseases. In both these epidemics the disease was almost always confined to the negro population, and thus in one prominent character very unlike the spotted fever which recognizes no color, class, age, or condition in life.*

* In December, 1812, a malignant typhus broke out at Camden, opposite Philadelphia. The disease appeared to possess characters different from ordinary typhus, and was extremely fatal under the system of treatment then pursued—evacuant.

When Dr. E. Strong's work on Spotted Fever appeared, the physicians were impressed with the idea that their malignant typhus was either a variety of, or a close analogue of, the fever described in that book. They thenceforth adopted the treatment he recommended—stimulants—with success.

In February, 1813, the same disease appeared in Philadelphia, first in the

Indeed, we might say that this fever is almost exclusively peculiar to New England, so rarely has it been seen in recent times outside of those States. From what I learn from the physicians of Newport, it would seem that they have known the disease to have prevailed latterly along with scarlatina and typhoid fevers in the towns and counties adjacent. Very likely this is so, and many cases may have been taken for typhoid fever, pneumonia, &c., and reported as such, or again, as anomalous cases of fever, as in the instance of Dr. D. Crary, of Hartford (see *The American Journal of the Medical Sciences* for January, 1863, p. 146). An old practitioner from Maryland recognized the disease at once, in the first case that happened at Newport.

The army surgeons state that there were cases of this disorder during the winter, at Portsmouth, Va., Annapolis, and Washington, among the U. S. troops. A limited but fatal epidemic of the same occurred in Centre and York Counties, Pa., in the month of March. Between the months of February and April, a number of cases showed themselves in Philadelphia, and its neighboring towns of Manayunk, Norristown, Frankford, Chester, and the Falls of the Schuylkill. At Newport, Rhode Island, seven cases occurred among the midshipmen billeted on the school ship in the harbor, and on diligent inquiry, Assistant-Surgeon Rickets could learn of no well authenticated cases in the town. A quack asserted that he had had two under his charge; both died.

These are, so far as I can learn, the limits of the disease as it prevailed last winter, though I am inclined to believe that many cases presented themselves both in the towns and counties of the New England States, New York, and Pennsylvania.

It is stated that the spotted fever first appeared in this country at Medfield, Mass., in 1806, and soon after in Connecticut. In 1810, it prevailed in the county of Worcester, Mass., with unexampled mortality, baffling the best endeavors of the physicians. In the autumn of 1812, it appeared among the United States troops at Greenbush, and other military stations, making

Northern Liberties, and spread in various directions over the city. At the same time the fever was prevalent in Frankford, Abington, Byberry, and in Philadelphia County, in Bucks County, and various parts of New Jersey. The disease was complicated with throacic troubles, and was called "the epidemic pneumonia" by the practitioners in the various counties of the latter State. Several army surgeons, then in the neighborhood of the disease, recognized it as the spotted fever of New England. See their letters in the *Med. and Philosophical Register*, Vol. III., page 491.

In the *Eclectic Repertory*, Vol. III., page 542, there is an allusion to the disease as it occurred in Philadelphia and Camden, in 1812-13.

great havoc among them. So alarming, indeed, had the disease become that the counsellors of the Massachusetts Medical Society appointed a committee consisting of Drs. Thomas Welsh, James Jackson, and John C. Warren, to make all possible inquiry and investigation into its history and treatment. Their able, elaborate, and judicious report occupies a place in that Society's reports (Vol. I.)

During the winter and spring of 1813, it was prevalent and extremely fatal among the inhabitants of Vermont, in the upper part of the State of New York, in several inland towns of Massachusetts and Maine, assuming a number of treacherous shapes, and extremely mortal. Boston suffered at the same time, though in this city the disease affected principally the new levies of the United States troops.

The frequent appearance and fatal character of this disease drew forth several ably written papers on the subject. See report above mentioned, also a paper by Dr. Thomas Paige, of Hallowell; Treatise on Typhus Syncopalis (spotted fever), by Thomas Miner, M.D.; Gallup on Epidemics; Thacher's *Modern Practice of Medicine*, Boston, 1826; Thomas' *Practice of Medicine*, by Prof. D. Hosack, N.Y.; Dr. E. Strong on Spotted Fever, Mass. Med. Society Communications, Vol. II., &c.

From these various works it would appear that the disease was well understood in all its protean forms, and remarkable success followed its treatment.

Dr. Paige, in the paper above mentioned, describes four varieties of the disease. 1st. That which principally attacks the brain. 2d. The spurious *peripneumonic* form characterized by pain in chest and oppression of breathing, with cough and expectoration of viscid, dirty-brown matter, and in some of the most malignant cases, blood completely dissolved. 3d. Where the disease was directed to the stomach and bowels, producing cholera morbus or colic. 4th. When the extremities powerfully suffered with coldness, numbness, and pains. In all these different varieties one common type was observed as their basis, though veiled by the various modifications; and the same treatment was mainly successful.

So that it appears these local phenomena often draw away the attention of the physician from the main disease, spotted fever, as happened in Case VII., where the pneumonic symptoms were the most prominent, and this mistake was particularly apt to happen at this early period, the practitioners not being in possession of our present precious method of physical diagnosis; thus, what wonder to see the disease described under the various titles of peripneumonia typhoides, malignant pleurisy, &c.? So that, really, it was spotted fever that was alluded to by Dr. Hugh Williamson, as occurring in North Carolina, in 1792 (see *Medical Repository*, first series, Vol. II.); and the same is true of the epidemic which prevailed in 1749, in Rhode Island, and reported by Dr. John Bard, in *Med. and Philosoph. Register*, Vol. I. This is perhaps the earliest account we have of the spotted fever, as it prevailed in the United States. There is, indeed, no reasonable doubt but that the typhoid pneumonia, which has prevailed from the earliest period of our history at various times, and in various sections, is identical with spotted fever, for says Dr. Thacher, op. cit.: "According to its various symptoms and forms, this pestilence has been termed bilious peripneumonia or typhoid pneumonia. In some of its appearances and forms it may be identified with the petechial fever above mentioned, but if it be a distinct disease, there is an obvious and close analogy in their nature and character." Again, Dr. Hosack, in the Appendix to Thomas' *Practice of Medicine*, says: "This disease (peripneumonia typhoides) is not a 'new calamity,' an 'unknown epidemic,' as it has been represented by some writers; on the contrary it has been well described by Sauvages (see *Nosologia Methodica*, Vol. I.), Huxham, and others. The causes of the disease are no less compound than the disease itself. The local inflammatory affections are probably occasioned by the sensible changes in the atmosphere, while the typhoid character of the disease is derived from an epidemic constitution of the air, the same which has given rise to the typhus petechialis, or spotted fever, which has prevailed for some time past in our Northern and Eastern States, and which is doubtless a similar disease, with the exception that the present epidemic is implicated with symptoms of local inflammation of the chest, brain, or throat, &c., the effect of the present cold season of the year."

Dr. John Huxham described peripneumonia typhoides in the year 1759,* and Sydenham in 1680,† nearly two centuries ago; and the latter date is probably the earliest European account we have of spotted fever or peripneumonia typhoides, as it was then called.‡ Our medical fathers have given no description

* See Huxham's Works—De aire et morbis epidemicis.

† See Thomas Sydenham, M.D.—Opera omnia. Schedula monitoria de novæ febris ingressu, page 486; et Cap. v., page 550—De Febre stationaria ab anno 1635 ad 1690.

‡ Some writers have stated their belief that Cullen and Hoffman have also witnessed the disease, the former alluding to it as a synochal fever, and the latter as a catarrhal fever. Boerhaave and Lieutaud have imitated Sydenham in their allusions to the Febres Petechialis.

which to our apprehension embraces it, though we have no positive proof that it did not happen in the earliest times.

From all this, we conclude that spotted fever has prevailed at various times both in Europe and America, under titles expressive of some of its numerous local complications or forms, and thus giving rise to errors in the recognition of the nature of the various epidemics leading to the separation of diseases essentially the same, and ignoring the identity of the constitutional malady common to them all.

*Symptoms.*—The advent of spotted fever is marked in different cases by an exceeding variety of symptoms; scarce two cases resemble each other. I shall first give a resumé or enumeration of the morbid phenomena presented by the cases which were seen at Newport, and afterwards submit the cases themselves in detail.

Patients are sometimes suddenly arrested in their employment or pastime with intense headache and delirium; but more commonly the disease begins with shifting pains in the extremities and joints, headache often of the most atrocious character, nausea, or vomiting, along with a chill, which last, however, soon subsides, and the characteristic delirium and dulness set in. The delirium varies in intensity, occasionally furiously maniacal, generally moderate and quiet; there is extreme restlessness and jactitation. The sensibility of the whole surface is sometimes so unwontedly increased that the patient cannot even bear to have his hair touched. There is generally remarkable prostration of strength, and the limbs seem paralyzed, and are numb, and in some cases even insensible; there is deafness, dimness of sight, or even complete loss of vision. A few have convulsions and opisthotonos. The tongue is moist, yellowish, or brownish, never like the red, chapped, beef-like tongue of typhoid. The pulse small, even thready; sometimes extinct in very malignant cases, irregular, or intermitting; skin cold, and occasionally of a deadly pallor, and like polished marble; eyes glassy, and the pupils irregular in their action, sometimes contracted, then suddenly dilated. When reaction takes place, the pulse becomes fuller, skin warmer, and then there is marked uneasiness, the patient tossing himself in every direction with delirium; these symptoms remaining three days, the patient may be restored to convalescence, or the disease may advance into stupor, and come to a fatal termination.

The intestines appeared in general to be exempt from the effects of the disease, except in Case 6, where there was obstinate diarrhœa; the others occasionally required a mild aperient.

The bladder gives sometimes great annoyance, and hematuria is encountered from the very beginning. Profuse perspiration took place in one case, possessing a peculiar mawkish smell. One prominent symptom in Case 7 was inability to swallow.

One of the most peculiar marks of the disease is the *eruption*, which may occur in all stages of the disease, and in three of the cases below detailed it made its appearance on the first day, on the second day in three, on the tenth in one. The spots assumed the form of small, round ecchymoses of various sizes, from the head of a pin to the size of a split pea, of a light red color, like the bites of fleas. As the case advanced the splotches increased in size and coalesced, forming larger ones, or, properly patches, and in bad cases assuming a livid or purplish color. Again, the form was that of reddish streaks, as if caused by striking the parts with a bundle of twigs. In all cases the eruption was even with the skin, and appeared first upon the extremities, generally the upper, and then over the face and trunk. The duration of the spots varied, sometimes disappearing in two or three days, at others holding on for a couple of weeks, and then gradually disappearing as convalescence set in; or becoming larger and deeper on approaching death; and when this event happened, they resembled bruises, were very distinctly marked, and those previously quite light or almost imperceptible were readily observed. Most of the authorities upon this subject have generally stated that the eruption occurs throughout this disease in some of its varying degrees of size and colors, while on the other hand there is not wanting others who assert it to be only present in one out of six cases. All the Newport cases presented it, as will be seen below.

The passive hæmorrhagic character of the eruption was well understood by those who saw so much of it between the years 1810–18. Dr. Miner has observed that common typhus may prevail along with this disease in the same season, but its characteristic symptoms, eruption, &c., suffice to prevent errors in diagnosis.

The above resume of symptoms was drawn from the seven cases which I now append, and the statements of those who hourly watched the patients from the beginning of their illness to its end. The cases are described in the order in which they broke out upon the school ship. I should here remark that there are two vessels lying in the harbor of Newport attached to the Naval Academy, and used as schools for practical instruction of the midshipmen. The students occupied them as quarters, and slept in hammocks, in the usual manner of sailors, between decks. Those ships were always kept in the most excellent hygienic condition, as regards cleanliness and ventilation. For the latter, the Academy is much indebted to Surgeon James C. Palmer, U.S.N., whose suggestions as to the arrangement of steam pipes in the holds were carefully carried out by the superintendent of the institution with a most unlooked-for, but yet truly gratifying success, exceeding the most sanguine wishes of their originator. The spotted fever occurred upon only one of the vessels, and as soon as the patients showed symptoms of illness they were removed to the shore hospital, also under charge of Surgeon James C. Palmer, so that there was universal concurrence in the opinion that a residence upon shipboard had nothing to do with the origin or extension of the disease.

Case I. F. J. S., midshipman, aged 16, born in Virginia, was placed on the sick list January 15, 1863, complaining of sore throat with slight headache, for which some simple remedy was prescribed. Later in the day he complained of severe headache, fever, and delirium; there were jactitation and restlessness, tongue brownish. During the night these symptoms became more severe.

*Jan.* 16. This morning there is less headache, and though restless, there is no jactitation. An eruption has appeared on the legs and arms, in some places of the bright blush of erythema, in others livid and as large as a pea, the result of extravasation of the blood; there are also some dark colored pimples. Tongue brown, and the teeth covered with sordes. Yesterday evening his bowels were opened by a mild purgative, and a diaphoretic mixture with an opiate given. This morning cold water was applied to the head, and weak toddy given to support the strength, the typhoid condition being decided, with passive extravasation in the cutis.

17*th*. Patient in a drowsy condition, but when addressed answers promptly, and seems in full possession of his intellect. The livid spots are disappearing from the arms, but diffused blotches remain upon his hips; no fever; tongue now white in its middle, and red at the tips and edges. Three small pustules made their appearance upon the right leg.

18*th*. The spots disappearing; delirium continues, though when aroused converses lucidly whilst engaged in conversation. No excitement of pulse, complains of much pain in the joints. Tongue covered with a thick coating of brownish fur, the teeth with sordes. Bowels moved by an aperient; apply a blister 3 inches by 6 inches to back of neck, ice to head. Brandy toddy and milk. ℞.—Pulv. Doveri, gr. x.—hora somni. 7, P.M., tenderness in right iliac fossa with tympanitis.

*19th.* Slept some last night; delirium continues, and he passes his urine involuntarily. The *eruption* almost entirely disappeared, except the red patches upon his hips; pulse 145; extreme tenderness in epigastric and iliac regions; tympanitis. Teeth covered with sordes, and the tongue very brown and dry; debility extreme; face flushed; continued ice to head, sponge the surface with tepid water; dress blister with cerat. sabinæ; milk punch. ℞.—Ammoniæ carb. ʒj; mucilag. acaciæ, syr. tolutani, āā f℥ij.—M. A teaspoonful every hour.

*20th.* The fever continued to progress all day yesterday until his pulse beat 163 a minute; became comatose finally, and died at 11.20, P.M. No *post mortem* could be had in this case; anxious and distressed parents stood around the beds of most all of these young gentlemen from the beginning to the end of their disease, and took charge of their bodies when dead.

CASE II. L., midshipman, aged 17; born in N.Y.; admitted to the list January 28th, complaining of pain in the limbs, calves of the legs. The lower limbs present an eruption like the bites of insects, and he is inclined to believe that the roaches, with which the ship ("Santee") swarms, have bitten him. The spots are of an ecchymotic character, even with the skin, and of various sizes and tints, from pale red to livid. There is no other evidence of disease, whatever.

*Jan.* 29. Patient has still the same marks upon the logs, but nowhere else; back of the right hand swollen and slightly red; calf of the left leg swollen and painful, but not reddened. Pulse and tongue natural, in fact there is no evidence of disease anywhere, except the ecchymotic spots, which, under other circumstances than the present, would be taken for flea-bites, and not excite solicitude. Ordered, ℞.—Potass. chloras. ʒss; acid hydro-chlor. fʒj; aqua dest. f℥viij.—M. A tablespoonful every third hour. Bowels have been freely opened. Milk toddy.

*30th.* Spots on the legs fading; the swelling of right hand, which occurred during the night, diminishing; tongue clean; pulse natural in frequency, but too compressible; tenderness to the least touch all over the body, but particularly in the epigastric region, around the umbilicus, and in the right iliac fossa. No cephalic symptoms whatever. Stomach excessively irritable, and vomiting of a purplish-colored matter. The chlorinated solution was suspended, and tr. chloroform comp. fʒj after each emesis. ℞.—Quiniæ sulp. gr. ij; strychniæ gr. 1-60th—M. Every four hours. Blister 6 inches by 4 inches to epigastrium continued sufficiently long to produce only a rubefacient action. P.M., was removed to the hospital in Newport, where immediate improvement set in. No vomiting from 1 to 10 P.M.

31*st.* Well-marked improvement; pulse natural, both in frequency, force, and volume; petechiæ scarcely perceptible; slight tenderness to touch over abdomen; countenance natural; sponge with tepid water dashed with the liquor sodæ chlor.; continue strychnia every three hours. ℞.—Ol. ricini f℥ij; ol. terebinthinæ gtt. x.—M. Take. Raw oysters, jelly, and milk punch—quiniæ sulph. gr. ij every sixth hour. P.M. Has passed the day favorably, except that he had an attack of vomiting, which was relieved by sinapism.

*Feb.* 1. Symptoms all favorable; bowels moved; pulse natural; tenderness scarcely perceptible; continue strychnia and quinia. ℞.—Cerii oxalatis gr. ij, every third hour.

3*d.* Is decidedly convalescent, requiring no more medicine; continue nourishment.

6*th.* Gaining strength; out of bed.

14*th.* Continued to improve up to the present, and was discharged cured.

Case III. W. K. B., midshipman, aged 17; born in Connecticut; was admitted to the sick list March 16th. Early in the morning small red spots, even with the surface, were observed upon the face and wrists, and were carefully watched; during the evening similar petechiæ were seen upon the lower extremities. Fever and delirium also set in at this time with extreme restlessness; was brought from the ship and lodged in the hospital. Ordered, ℞.—Quiniæ sulp. gr. xij; strychniæ gr. ss.—M. et ft. pill. No. iv, one every four hours.

*March* 17. Has been delirious and in constant motion all day. Cut off his hair and applied cold to head. Continued same medicine as yesterday without the quinia.

18*th.* The patient is rather more comfortable, and has lucid intervals in the delirium; pulse soft; tongue natural; passed his urine, and had a motion of the bowels. Continue strychnia; apply blister to neck; cold to head.

19*th.* Abatement of all the symptoms. The strychnia was omitted yesterday evening on account of the occurrence of cramps in the gastrocnemii muscles. Repeat the medicine twice to-day.

19*th.* Had a paroxysm of fever towards evening; delirium pretty constant, though he recognizes his friends and answers correctly when spoken to. Bowels not moved. ℞.—Ol. ricini f℥ij; coughs a good deal, and expectorates bloody mucus; fauces have been inflamed from the beginning. No abnormal sounds can be heard in the chest. Puts his hand frequently upon the right hypochondriac region as if he suffered pain there. Apply blister 4 inches by 6 inches over this region until the skin is reddened. Milk punch during the night. Continue the strychnia. Pulv. Doveri gr. viij, hora somni.

*20th.* Passed last night comfortably, generally in a sound sleep. Pulse less frequent, softer, and more feeble; cough much abated; tongue more moist. ℞.—Pulv. Doveri gr. v, ter in die, strychniæ gr. 1-32 t. d. Diet, oysters, milk punch, &c. Dress blister on the neck. P.M. The febrile exacerbation less violent than yesterday. Continue treatment throughout the night. Sponge surface with a mixture of equal parts of vinegar and water.

*21st.* Passed a good night; urinated twice, and is inclined to complain of trouble about the bladder. Suspend strychnia. R.—Spt. æth. nit. f℥j, ter in die. Dress blister. Milk punch, oysters, Scotch ale, and quiniæ sulph. gr. ij, ter in die.

*22d.* The symptoms more favorable. R.—Tr. ferri chlorid. gtt. viij, ter in die; other medicines suspended; continue supporting diet, and give at bedtime, R.—Morphia sulph. gr. ¼.

*23d.* Recovering slowly but yet decidedly. Continue iron and diet.

*25th.* Convalescent. Continue diet and the tincture chloride of iron.

*27th.* The patient had to-day an accession of fever, is quite restless, with occasional opisthotonic convulsions; suspend the iron and resumé the strychnia in same dose three times a day. Milk toddy, broth. R.—Morph. sulph. gr. ¼, hora somni.

*28th.* The excitement of yesterday subsided. Nourishing diet; suspend medicine.

*29th.* Comfortable; diet same.

*April* 1. Again convalescent; diet as formerly.

*3d.* Condition good; has profuse perspiration. R.—Acid. sulph. aromat. gtt. x, ter in die; ale, nutritious diet. P.M. Though doing so well up to this morning, he had been kept in a state of nervous agitation all day by the minute cares and extreme solicitation of his mother, who is sometimes unable to restrain her grief. His father arrived at 4, P.M., and though he did not at this time appear excited, in a few moments afterwards he fell in a paroxysm of convulsions, which continued, with short remissions, till 9.30, P.M., and then yielded for about one hour. Mustard poultices were intermittently applied; cold to head, and his feet put into hot water; an enema of turpentine was also given. The second paroxysm yielded at 2 A.M. next morning, and left him insensible. The pulse varied all night in force, frequency, and regularity. ℞.—Tr. valerian f℥ij, Brandy ℥ss during the day.

*4th.* Somewhat more tranquil up to noon, and partially restored to sensibility; pulse fuller. Repeated turpentine enema; wine freely; cold to head, and hot water to extremities continued. At 6 P. M. had profuse *black vomit*, which kept up to the last. He expired at 8.30 P.M. It should be remarked that this young man came from Middletown, Connecticut, and had a few weeks before convalesced from scarlet fever. His family physician observed that this season all fevers in that place had a disposition to the typhus type, and I believe also said there were or had been spotted fevers there recently. He had been on board ship only a few days when taken sick.

Case IV. D., midshipman, aged 16; born in Maine; was admitted March 29th. Was seized suddenly, and brought early from the school ship "Constitution" to the town hospital. Has headache, vertigo, and stupor; pulse thready; ecchymotic spots upon lower limbs. ℞.—Strychniæ 1-32 t. d., blister to neck; wine.

*March* 30. Stupor and vertigo less; pulse somewhat stronger and less frequent; fuller; tongue moist; headache still continues. Brandy toddy, beef-tea, and strychniæ. gr. 1-32. To allay the nausea which has been very distressing. ℞.—Tr. chloroform. comp. gtt. xxx.

31*st.* Continues to improve; reaction complete; pulse increased in strength and less frequent; cephalic symptoms gone. Continue medicine and diet.

*April* 1*st.* Partial opisthotonos occurred last evening, the strychnia was accordingly suspended. Pulse irregular and feeble, but brought up by the free use of brandy.

2*d.* Opisthotonos relaxed during the night, but he was excessively restless until tranquilized by an opiate enema; continued to lie upon his face until morning; when he awoke he spoke but once, "it hurts," and was turned over upon his back. Brandy and stimulants were used freely without avail; he breathed his last at 9.10 A.M.

Case V. G., midshipman, age 14; born in New York; admitted to the sick list April 8, 1863. At half-past ten o'clock last night he complained of sore throat, and had some fever and headache. The weather was bad, and prevented his immediate removal to the Naval Hospital in town, so he was detained on board until morning, having taken, during the night, spt. æther nit. When arrived at the hospital, he had stupor from which it was difficult to arouse him, but at these times he answered sensibly; headache; pulse thready, almost imperceptible; death-like pallor; extreme debility; arms and legs dotted with minute ecchymoses; and passed the urine and feces involuntarily, the latter profuse, black, and intensely fetid. Stimulants were administered from the moment of his arrival, ammoniæ carb., sherry undiluted, milk toddy, and sinapisms to abdomen and extremities; these remained on a half hour before reddening the skin. The pulse did not rise under treatment. At 10 A.M. began taking the following formula: ℞.—Strychniæ gr. j; aquæ destil. f℥iv; acid. sulph. aromat. q. s. ft. sol. A tablespoonful every fourth hour. Also R.—Quiniæ sulph. ʒj; tr. chinchonæ comp. f℥ss; acid. sulph. aromat. q. s.; aquæ f℥j. A tablespoonful every sixth hour. Frictions with tr. capsicum and chloroform to spine and surface. P.M. Had another evacuation, less profuse but of the same character as the first. Later in the evening some signs of reaction; pulse sensible to touch; great jacitation; pallor yielding, and there is even a tinge of redness on the cheeks; frictions kept up.

*April* 9. After 2 A.M. this morning, the patient became furiously delirious, and died at 7.48 A.M. Frictions with hot water, &c., and the internal administration of stimulants were perseveringly followed up to the last moment.

CASE VI. T. T., midshipman, age 17; born in New Hampshire; was admitted April 13th, complaining of pain in the glans penis; and of passing blood; warm hip-bath, and cups to spine near kidneys were ordered. R.—Spts. æther. nit. f℥j ter in die; pulv. Doveri gr. x, hora somni.

*April* 14. No material change in his condition since yesterday. Continue treatment.

15*th.* Hæmaturia much diminished, and is now laboring under severe catarrh; ordered magnesia ʒj now; afterwards he was ordered quiniæ sulph. gr. ij, ter in die, and five grs. of the pil hydrarg. to be taken once during the day.

16*th.* Improving a little; urine slight, tinged with blood; nausea and pains of a rheumatic character in extremities; continue medicine as yesterday. To relieve nausea, R.—Tr. chloroform co. gtt. xxx, ter in die.

17*th.* Bowels constipated; enema and R.—Hyd. chlor. mitis gr. v; pulv. rhei gr. x.—M. Urine somewhat discolored. Spt. æther. nit. and infus. lini.

22*d.* Up to this time has improved; no blood in urine; general condition better; pulse stronger; bowels somewhat constipated, for which was ordered a saline aperient; petechial spots have appeared on arms and legs.

23*d.* The parotid on left side has enlarged considerably. Apply unguent. iodinii. ℞.—Potass. iodidi gr. viij, tr. gentian comp. f℥j, ter in die.

24*th.* Enlargement of right parotid gland this morning; the left very large. Continue iodine externally and internally.

25*th.* Both sides neck very much swollen, otherwise doing well. Continue medicine.

26*th.* Swelling declining on left side, and rather larger on the right; colicky pains sometimes severe. Omit iodide potass. ℞.—Tr. chloroform comp. gtt. xl, at once, and repeat this medicine in doses of 20 drops pro re nata.

29*th.* Tumor on left side pouting, and declining on the right. Continue potass. iodid.

30*th.* Opened the abscess on left side at the angle of the jaw, and let out a large quantity of pus, with immediate relief. Apply poultice, and continue medicine; supporting diet.

*May* 1. Opened abscess on right side, and poulticed.

2*d.* Both abscesses discharging profusely, and passed stools, tinged with blood, during the night. ℞.—Calomelanos gr. v, ol. ricini ℥j; M.; also tr. chloroform comp. gtt. xxx, twice.

3*d.* Bowels being loose last night, he took ℞.—Creta ppt. ℥j, pulv. Doveri ℥ss, ft, chart. No. j, and then R.—Ext. nucis vom. gr. ij, pulv. opii gr. vj, M. et ft. in pill. No. vj. One every six hours. Diet supporting.

7*th.* Improving gradually, but his bowels keep rather loose, though the last two stools are natural. The abscesses are discharging less. Suspend all medicine, ordered one pint Scotch ale daily, and good nutritious diet.

8*th.* Small stools and numerous. R.—Plumbi acet. gr. vj, pulv. opii ij. M. ft. pil. viij. One every three hours; milk toddy, and warm diet.

9*th.* Pulse small, and 140 a minute; ecchymotic streaks extending from both groins upwards upon the abdomen; discharge from abscess continues, and the diarrhœa is also frequent, but not large stools; these were checked for some hours by an enema of tannin. During the forenoon fell in heavy naps and snoring stertorously. Milk toddy, beef-tea, acid. nit. gtt. j, in each draught of beef-tea.

10*th.* Pulse 130; general appearance improved, though he is still very low. Supporting treatment. P.M. R.—Pil. hyd. gr. v.

11*th.* No evacuation since 3 P.M. yesterday. The enema of tannic acid has been repeated several times. Continue beef-tea and nitric acid, with other nourishment.

*12th.* Has diarrhœa again to-day, but was promptly checked by enema and opiates. Passed the night pretty comfortably.

*13th.* Pulse 100, and whole appearance indicating an improved condition of the patient, bowels loose; gave an enema after each evacuation.

*16th.* The patient remained about same up to this evening, when he discharged a quantity of coagulated blood from the bowels, and a general hemorrhagic disposition prevails. Continue to use nourishing and supporting aliments and stimulants.

*24th.* The patient continued in this debilitated and uncertain condition until to-day; there now seems to be a decided improvement; pulse 100, stronger and fuller. Continue same line of treatment.

*27th.* Convalescent. Continue treatment.

CASE VII. V., midshipman, age 15, born in California, was admitted April 30, complaining of sharp pain in the side, pleuritic in character; has had his feet wet; dry cups to side, all bleeding, either local or general, being contraindicated. Apply a blister 6 in. by 6 in. over seat of pain. R.—Potass. et antim. tartrat. gr. $\frac{1}{4}$, every third hour.

*May* 1. Febrile movement moderate. Dress blister; ecchymotic spots upon legs and arms. R.—Spts. æther. nit. f℥j, ter in die.

*4th.* Up to yesterday has had fever at intervals, but to-day it has been constant; no delirium. R.—Quiniæ sulph. gr. iij, bis in die, pulv. Doveri gr. viij, hora somni. No pain; the sputa tinged with blood. Continue quinia, effervescing draught, spts. æth. nit.

*5th.* But little blood in sputa; little cough; pulse weak; complains of sharp pain at a certain point on the œsophagus, which makes him averse to swallowing. Suspend all medicine; give nourishing food in small quantities at a time, and frequently.

*6th.* Swallows more easily. Continue diet.

*7th.* Improved a little; pulse 60, and peculiar, with long intermissions; the pneumonia, of which there were physical signs, has yielded to the treatment pursued; little fever. Add to each draught of the beef-tea acid. nitric. gtt. j.

*10th.* Less difficulty in swallowing; no fever; ecchymotic spots gone; little or no cough. Continue medicines.

*14th.* Up and out of doors; has been taking following formula: R.—Quiniæ sulph. gr. xvi; tr. gentianæ comp. f℥j; aquæ f℥iij.—M. A tablespoonful every third hour.

*Causes of the Fever.*—It was suggested, a long time ago, by some of the New England physicians, that spotted fever resulted from the use of spurred rye, but this opinion was manifestly untenable, inasmuch as the disease prevailed among those against whom no such a cause could be operating. In a short time it became a settled opinion that its prevalence, like other epidemics, depended upon a peculiar state of the atmosphere, and the predisposition of the people being favorable to its operation. Yet they recognized as exciting causes intemperance, exposure to cold and wet, fatigue, anxiety of mind, and fears. So great is the latter an exciting cause, that Thacher remarks, "that the most fatal consequences have been known to result from the influence of horror and fear. The terrific name spotted fever, or cold plague, its well-known fatality, the tolling of bells, its frightful visage, the weeds of mourning, and the tears of sorrow, wonderfully conspire to induce a morbid state of the system favorable to the reception of the disease, and tend more immediately, perhaps, than any other causes to multiply the instances of mortality." The disease is recognized by all *not to be contagious*, and the epidemic at Newport showed not the slightest disposition to spread.

*Its Duration.*—In some cases the disease produces death in five or six hours, in others runs on for three or four weeks before a fatal termination. Case III. appeared to have died after seeming convalescence had been established. The greatest number die on the third and fourth days. In mild cases convalescence may be established on the third day, and, indeed, some extremely bad ones mend rapidly from that period. Dr. Miner states that in many of those cases which were neglected or treated with evacuants, a peculiar and usually irreparable sinking and exhaustion occurred on the third, fifth, or more commonly on the seventh day.

*Age and Sex.*—All classes, sexes, and ages, from one year to seventy, are its indiscriminate victims, though I am unable to find any reliable statistical matter bearing upon these points, in the works of those who have generally made these statements; indeed, we are led to believe that the rich and the poor, those living in sparely inhabited districts, and those in towns and cities; those in densely crowded houses, and those in large, airy mansions, were alike subjects of the disease. Locality may have some influence upon it, for in the early epidemics the disease affected more particularly and more fatally the inland towns of New England, while those on the sea coast escaped, or had it in some milder form.

*Mortality.*—In New England the horror of the ravages of yellow fever had scarcely abated before spotted fever made its appearance, and was not less malignant and deadly than its predecessor. Prevailing more or less extensively in the interior of the country, and on the seaboard during the cold and damp months of winter and spring, this fever in some places on its first appearance was fatal to more than half those attacked; in other seasons and places the mortality was less, and under favorable circumstances only one in thirty or forty died. In Newport more than half died, and in Philadelphia and its environing towns, one out of every four or five cases proved fatal.

It appears that the mode of treatment has a vast deal to do with the result of this disease, for Dr. Miner mentions that two physicians, in the year 1823, had charge of 360 cases, of whom only twelve died, six adults and six children.

However, the mortality of the disease will vary with the season and locality, and observe the law of other epidemics in being more fatal at the beginning than at the latter periods of its epidemic occurrence. We might add this additional reason, when so much depends upon the prompt and energetic treatment of the fever, of its greater mortality during last winter than during the winter of 1823, the want of a sufficient acquaintance with its nature and treatment by the present generation of physicians, for to them it was a new disease, an unknown emanation from the box of Pandora.

*Anatomical Lesions.*—Unlike some of the essential fevers, this affection does not present any characteristic lesions, but simply such as result from an *altered integrity of the blood*, characterized by a disposition to escape from its vessels. In those cases examined from 1813 to 1816, the brain and its meninges were always found congested, effusion of serum into the ventricles and subarachnoid spaces. One author states he met with coagulable lymph in the lateral ventricles. Changes in the heart, pericardium, lungs, and pleuræ, indicating generally passive congestions, subserous effusions of blood in patches of small extent, occasionally inflammation. The stomach showed submucous spots of the same character, and contained black vomit or such fluid as noted in Case III. No autopsic examinations were had in the Newport cases, and thus much valuable information was lost. It might be well to observe here, that the medical officer in the Navy has much opposition to encounter in the pursuit of *post mortem* investigations, springing, in many cases, from deplorable superstition on the part of the sailor, and not unfrequently from the prejudices and narrow-minded developments of officers, more particularly those of the old school, whose travel and experience would seem to have circumscribed instead of expanding their liberality and common sense.

*Diagnosis.*—When this disease first broke out on the practice-ship Constitution, the first case caused some speculation and surmise as to its nature, but the correct diagnosis was readily arrived at in the second case. Dr. Miner truly observes, that "there may be with the inexperienced some hesitation as to the nature and name of the complaint; but upon the whole there is less liability to mistake than in the diagnosis of any other acute fever with which we are in the habit of meeting in the ordinary course of practice. Dysentery, cholera, cynanche, catarrh, cough, pneumonia, measles, rheumatism, gout, and even common typhus are often complicated with it; yet there is always some prominent symptom by which it may be determined when the general affection is that of typhus syncopalis" (spotted fever.)

The suddenness of its attacks, the prominent severe headache and pain in the limbs, from the very beginning, with delirium, stupor, and coma, and the occurrence in two or three days of an eruption, mark it at once as peculiar and distinct from the few diseases with which it could only be possible to mistake it. The countenance is expressive of extreme suffering and anxiety, and in some cases of a dull, sallow hue, quite characteristic. It will be seen that in some of the cases above detailed, soreness of the throat was a prominent symptom, and this might lead one in the beginning of the affection to prognosticate scarlet fever, but the latter has an altogether different course, with an exanthem quite distinct from the ecchymoses of spotted fever.

Dr. Palmer was inclined to regard his first case as typhoid fever, but the rapid course of the disease, the early and peculiar eruption upon the skin, and general character of the delirium, so different from typhoid, led him to correct his diagnosis to petechial fever immediately.

I heard of a medical gentleman who felt quite sure the first case of spotted fever, that came under his care, was one of small-pox.

*Prognosis.*—The prognosis in this malady should always be guarded, and its epidemic character as to malignancy, and its secondary complications always kept in view. From the histories of the New England epidemics it appears that in those seasons when the disease showed grave cerebral complications more prominently, the prognosis was bad, and, on the other hand, it was more promising when only the organs of the chest and abdomen seemed to bear the brunt of the attack. In the first class of cases some of the patients were comatose almost from the very inception of the fever, and required speedy and active treatment to afford them even a chance of life. Miner describes a peculiar kind of thoracic functional derangement, irregular in character, "the inspiration occurring only at intervals of several seconds, and being usually long and full, while the expirations were sc short that the breath was parted with instantaneously. This condition, in combination with sinking, was often the first warning of danger in the insidious cases, and it was almost invariably irremediable."

Those patients who escape to the third or fourth day, with proper treatment, have encouraging chances of recovery.

*Treatment.*—In the treatment of this disease most all experienced physicians avoid blood-letting, and some condemn it in all cases. Dr. Page says that in the year 1816 he attended 220 cases of spotted fever, and bled but once to the extent of 8 or 10 ounces—a robust man, and even it might, in this instance, have been avoided. Dr. Miner holds nearly the same language, and states, "it should be observed as a rule to avoid anything that might *tend to waste the vital powers.* Evacuations, if copious, invariably render the mild cases severe, and the severe ones fatal. Probably more than three-quarters of the fatal cases were the consequence of spontaneous or factitious purging or vomiting."

Not one of the Newport cases could have been bled without dangerous, if not fatal, results.

*Purgatives.*—All energetic purgatives are likewise condemned, and one of the authors who has given the clearest account of the therapeutics of this disease, objects to the use of these medicines altogether until after the third day, when the mildest of them may be employed, as castor oil, rhubarb, &c., and along with a host of other practitioners, speaks highly of an injection of milk, salt, and sugar. Some patients were known to have died while under the operation of a dose of calomel and jalap.

*Emetics.*—Emetics of ipecac and sulphate of copper engaged the confidence of most practitioners when there was "a foul state of the stomach."

*Epispastics.*—This class of remedies always ranked high as therapeutical means in the treatment of this malady, and were always ordered early in the disease, and as near the part most affected as possible; and in order to obtain these speedy good effects, the skin should first be excited by friction with strong tincture of cantharides—"so highly beneficial are these effects,"

says Dr. Thacher, "that blisters ought to be applied in succession to the head and chest until the most effectual relief be obtained. In every case of considerable violence the head should be immediately shaved, and cold water and vinegar applied, while the back of the neck and temples are vesicated."

*Opium.*—Dr. Miner speaks of this drug as almost a specific, and I cannot do better than quote his own language: "A few cases imperiously required half an ounce of the tincture of opium in an hour, or half a drachm in substance in the course of twelve hours, before urgent symptoms could be controlled; and even some cases required a drachm in the same time. *All those patients whose symptoms were promptly met with opium invariably recovered.*"

*Arsenic*, in the form of Fowler's solution, acquired throughout New England, where the disease most prevailed, considerable reputation, and the most experienced physicians agreed in their expressions of confidence in its superior efficacy. The dose recommended was four to six drops every four or six hours, until its effects upon the system became evident by a peculiar sensation about the eyes.

*Strychnia.*—Dr. James C. Palmer, U.S.N., used, as he believes with great advantage, strychnia, beginning with the article early in the disease, and it will be seen that most of the cases above detailed were thus treated. Care should be taken that we should not confound the tetanic movements, a phenomenon of the disease, with those, the result of strychnia. I am inclined to think that the good effects attributable to that drug in the above cases resulted more from the quinia used in combination with it, and the stimulants employed at the same time.

*Stimulants.*—We come now to the most important class of remedies in the treatment of spotted fever, and those which bring us unmistakably immediately good results, saving patients from certain and impending collapse. Stimulants are applied in the usual manner externally, hot bricks, bags of sand, hot foot-baths, &c., billets of wood heated and applied to different parts of the patient's body placed between blankets. Frictions of the whole body with sweet oil have been highly recommended.

Among the milder internal stimulants we have hot tees made of sage, origanum, pennyroyal, peppermint, and the dwarf yew; more active than these, the volatile oils, particularly the oil of turpentine, both by the stomach and rectum.

Brandy and camphor form an excellent combination. Dr. Hall relates the case of a young married lady who was attacked by spotted fever after her first lying-in, and had mild delirium, which soon rose to the most violent fit of distraction, with supervening coma. In one hour 40 grains of camphor, and 180 drops of laudanum were given to her; and in the following three hours she took 400 drops more, a bottle of Madeira wine, and some brandy; immediately after which she began to mend, and gradually recovered, contrary to the expectations of all her friends. In another case, where coma had set in, he gave in six hours 500 drops of laudanum with a quart of wine, and nearly as much brandy; the patient recovered.

Quinia and bark were almost always administered towards the end of the disease, and followed up, when convalescence was established, by beef, mutton, and chicken.

I cannot do better than finish this summary of treatment by the observation of Dr. Hall: "That no disease requires more careful nursing, and perhaps none is more liable to relapses, and when severe relapses do occur, they are frequently dangerous, and often fatal; but are to be treated as new cases."

